# Role and Interpretation of Antifungal Susceptibility Testing for the Management of Invasive Fungal Infections

**DOI:** 10.3390/jof7010017

**Published:** 2020-12-30

**Authors:** Frederic Lamoth, Russell E. Lewis, Dimitrios P. Kontoyiannis

**Affiliations:** 1 Infectious Diseases Service and Institute of Microbiology, University Hospital of Lausanne, Lausanne University, 1011 Lausanne, Switzerland; frederic.lamoth@chuv.ch; 2 Clinic of Infectious Diseases, S’Orsola-Malpighi Hospital, Department of Medical and Surgical Sciences, University of Bologna, 40126 Bologna, Italy; russeledward.lewis@unibo.it; 3 Department of Infectious Diseases, Infection Control and Employee Health, The University of Texas MD Anderson Cancer Center, Houston, TX 77030, USA

**Keywords:** invasive aspergillosis, invasive candidiasis, mucormycosis, clinical breakpoints, minimal inhibitory concentration, therapeutic response, pharmacodynamics

## Abstract

Invasive fungal infections (IFIs) are associated with high mortality rates and timely appropriate antifungal therapy is essential for good outcomes. Emerging antifungal resistance among *Candida* and *Aspergillus* spp., the major causes of IFI, is concerning and has led to the increasing incorporation of in vitro antifungal susceptibility testing (AST) to guide clinical decisions. However, the interpretation of AST results and their contribution to management of IFIs remains a matter of debate. Specifically, the utility of AST is limited by the delay in obtaining results and the lack of pharmacodynamic correlation between minimal inhibitory concentration (MIC) values and clinical outcome, particularly for molds. Clinical breakpoints for *Candida* spp. have been substantially revised over time and appear to be reliable for the detection of azole and echinocandin resistance and for outcome prediction, especially for non-neutropenic patients with candidemia. However, data are lacking for neutropenic patients with invasive candidiasis and some non-*albicans Candida* spp. (notably emerging *Candida auris*). For *Aspergillus* spp., AST is not routinely performed, but may be indicated according to the epidemiological context in the setting of emerging azole resistance among *A. fumigatus*. For non-*Aspergillus* molds (e.g., *Mucorales*, *Fusarium* or *Scedosporium* spp.), AST is not routinely recommended as interpretive criteria are lacking and many confounders, mainly host factors, seem to play a predominant role in responses to antifungal therapy. This review provides an overview of the pre-clinical and clinical pharmacodynamic data, which constitute the rationale for the use and interpretation of AST testing of yeasts and molds in clinical practice.

## 1. Introduction

Early appropriate antifungal therapy is a key determinant for the outcome of invasive fungal infections (IFIs). While first and alternative therapeutic choices have been well defined for the most frequent IFIs, such as invasive aspergillosis (IA) and invasive candidiasis (IC) [1,2,3,4,5], or other less frequent IFIs (e.g., mucormycosis, fusariosis, scedosporiosis) [6,7], much uncertainty remains about the role and interpretation of antifungal susceptibility testing (AST). For some fungal pathogens, antifungal susceptibility patterns are well known with limited intra-species variability (e.g., *Scedosporium apiospermum*). For others, the significance of minimal inhibitory concentration (MIC) in predicting outcome is notoriously weak and AST is not routinely recommended (e.g., *Fusarium* spp., *Mucorales*). However, for *Candida* spp. and *Aspergillus* spp., the two most frequent fungal pathogens, emergence of acquired antifungal resistance is a concern and definitions of clinical breakpoints (CBPs) are needed for the distinction between susceptible and resistant isolates in order to inform appropriate antifungal selection. Both the Clinical and Laboratory Standards Institute (CLSI) and the European Committee on Antimicrobial Susceptibility Testing (EUCAST) are working on establishing and updating CBPs for fungi. However, this task is complex, as illustrated by the reassessment and changes of CBPs over time, some discrepancy in CBP definitions between CLSI and EUCAST, and the absence of CBP definitions for some fungus/antifungal drug combinations (Table 1).

The aim of this article is to discuss the specific challenges of CBP definitions for the most relevant fungal pathogens of IFIs and to review the current evidence of MIC/outcome correlations.

## 2. The Challenges of Fungal Clinical Breakpoints (Cbps) Definitions

**Figure 1:**
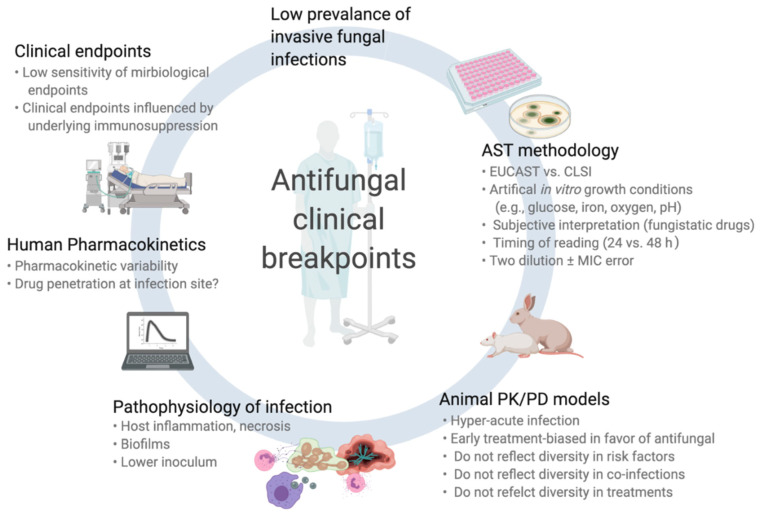
Challenges and pitfalls in the assessment of clinical breakpoints for fungi and antifungal drugs.

While often well established for antibacterials, CBPs for antifungals are associated with greater uncertainty. This is in part due to the relatively low prevalence of IFI (in particular mold infections) and some specific biological characteristics of fungal pathogens. In this section, we will review these specific aspects (summarized in Figure 1).

### 2.1. Antifungal Susceptibility Testing (AST) of Fungi

Some nuances of AST exist. First, the artificial in vitro conditions of testing by microbroth dilution method may differ considerably from the actual pathophysiological environment of IFIs. For example, invasive infections by molds affect mainly solid tissues (rather than biological fluids), have relatively low fungal inoculum (compared to the very high spore concentrations used in AST), and are often accompanied by tissue infarction and necrosis that might preclude appropriate drug penetration at the site of infection. In addition, the chemical composition of AST growth media differs from real pathogenic conditions regarding important elements for fungal growth (e.g., glucose, iron, oxygen, pH). Moreover, routine in vitro testing conditions do not take into account the possibility of biofilm formation (especially for *Candida* spp.).

Different AST methods are used in routine across countries or local laboratories. CLSI and EUCAST methods are recognized as the standard procedures and MICs derived from these methods are used for definitions of epidemiological cut-off values (ECVs) and CBPs [8,9,10,11]. CLSI and EUCAST procedures exhibit some notable differences (e.g., related to glucose content, spore inoculum, and reading interpretation), which may explain some differences between their respective CBPs. Moreover, these methods are manual and fastidious with an accepted margin of errors of up to ± two dilutions, which may considerably impact MIC classification and interpretation. As a consequence, many laboratories use commercially available microbroth dilution method (e.g., Sensititre YeastOne^TM^, Vitek-2^TM^) or alternative methods (E-tests, agar disk diffusion), which may result in significant differences in MIC results, despite relatively good essential agreements [12,13,14,15]. Important interlaboratory discrepancies have also been notified regarding AST of caspofungin for *Candida* spp., which resulted in the withdrawal of CBPs recommendations by EUCAST [16]. Another common issue with AST consists of the difficulties in MIC determination for some drug/fungus, which may lead to discrepant results as a consequence of different subjective interpretation from the reader. This is principally the case for antifungal drugs for which there is a fungistatic activity and a trailing effect (e.g., *Candida* spp. and azoles, *Aspergillus* spp. and echinocandins) or a paradoxical effect at increased concentrations (*Candida* spp. and *Aspergillus* spp. with echinocandins). Moreover, the timing of reading (24 vs. 48 h), which is not consistent across studies, may affect MIC determination.

Finally and most importantly, AST results for fungi are usually obtained after a significant delay due to their slower growth rate compared to bacteria (i.e., several days to one week). Their interpretation for patient management is not made in “real time” and their impact on outcome is therefore limited, since early and appropriate antifungal therapy is of paramount importance for success.

### 2.2. Animal Pharmacodynamic/Pharamacokinetic (Pk/Pd) Models

In view of the paucity of clinical data, murine Pk/Pd models are important for assessing the correlation between drug exposure/MIC and therapeutic response. These data are also taken into account for CBP definitions. However, an element of artificiality also exists in these in vivo models as it compares to the complex clinical scenarios of IFIs in humans. For example, much higher fungal inocula are administered to mice, an innately non-susceptible species to fungal diseases, and only following intensive immunosuppressive regimens (e.g., myelotoxic drugs and corticosteroids) in order to induce a quick and intense (rapidly fatal) infection. These conditions do not reflect the diversity of immunosuppressive and other co-morbid conditions in humans, the variety of IFI types and localizations (e.g., pulmonary vs. cerebral aspergillosis, candidemia vs. non-candidemic IC) and the actual timing and course of infection.

### 2.3. Pathophysiology of Invasive Fungal Infections (IFIs)

The distinct features of IFI (compared to bacterial infections) represent the most important aspect of interpreting MIC results for fungi. Mold pathogens are relatively infrequently isolated in culture and MICs are therefore lacking [17]. The diagnosis of IFI (in particular for molds) is complex and associated with some degree of uncertainty with a high rate of possible/probable infections and a lower rate of proven infections, which may bias outcome analyses towards later diagnosis when the fungal burden is high. The timing of diagnosis and initiation of antifungal therapy is also crucial and frequent delays in IFI diagnosis have a considerable impact on outcome. The localization and extension of infection may also affect the therapeutic response. For instance, drug penetration within the different organs commonly affected by IFI (e.g., lungs, brain, or skin for IA, blood, or peritoneal cavity for IC) may be quite different [18]. Most importantly, the outcome of IFI is highly influenced by non-pharmacologic parameters, such as host variables (type, severity and potential for recovery of underlying diseases and immunosuppression) or adjunct therapies such as surgical interventions.

The assessment of therapeutic response in IFI is also difficult. While objective criteria can be monitored in candidemia (e.g., clearance of blood cultures), the outcome evaluation of invasive mold infections essentially relies on radiological interpretation, which may be confounded by other infectious or non-infectious (e.g., sequela of surgery, inflammatory reaction) radiological patterns with frequent initial worsening of lesions at the time of neutrophil recovery. Moreover, the assessment of response for invasive mold infections requires prolonged follow-up (weeks to months) and overall survival may be affected by multiple intercurrent infections/events in these patients with severe underlying diseases, such as cancer.

The rarity of some IFIs makes that many clinical pharmacodynamic studies present very heterogeneous data pooling different type of IFI (IC, IA, and other IFIs) or different species within a same genus (e.g., *Candida albicans* and non-*albicans Candida* spp.) or different grading of IFI (proven/probable or possible, empiric treatment for suspected IFI without documentation). Moreover, uniform therapeutic approaches are needed for outcome analyses and IFIs often require multiple lines of different antifungal treatments or drug combinations. Because of the delay in culture results, initial antifungal therapy is usually empirical and then switched to targeted treatment.

## 3. Correlation between Mics and Outcome: Current Evidence

Determination of CBPs rely on ECVs derived from epidemiological studies, animal Pk/Pd models and, most importantly, clinical studies supporting the reliability of MIC in predicting outcomes. In this section, we will review the current evidence supporting a correlation between antifungal drugs MIC and clinical outcomes for the most frequent IFIs and for the three antifungal drug classes (polyenes, azoles, and echinocandins).

### 3.1. Invasive Candidiasis (IC)

IC is the most frequent IFI and consists of candidemia in a majority of cases, which means that there is a relatively robust set of data with documented pathogen/MIC and objectives parameters of outcome measurement, such as the clearance of candidemia. Based on these data, CBPs have been proposed by both CLSI and EUCAST for the most frequent antifungal drug/pathogen combinations (Table 1). However, most trials of candidemia, from which these data have been derived, have been performed in non-neutropenic patients. Data from candidemia in neutropenic hematologic cancer patients or from non-candidemic IC (e.g., hepato-splenic candidiasis, intra-abdominal candidiasis) are lacking [19].

### 3.2. Amphotericin B

Pk/Pd murine models of IC have shown that the C_max_/MIC ratio of amphotericin B (AMB) was the determinant predictor of outcome with a ratio ≥10 resulting in maximal fungicidal effect [20,21,22]. However, poor solubility of AMB at physiological pH and high degrees of protein binding of insoluble drug may create a low ceiling (<100 ng/mL) for concentration-dependent fungicidal activity in many tissues, resulting in accumulation of non-bioactive protein bound [23].

AMB is currently not a first-line treatment of IC. Analyses of MIC/outcome correlation are therefore scarce or relatively old. One study suggested a correlation for a MIC threshold of 1 mg/L [24], while another study derived from a large randomized controlled trial of AMB versus fluconazole did not found any correlation [25]. This lack of correlation was confirmed by a more recent study among 107 candidemic episodes [26]. The narrow range of MICs (0.25–1 mg/L) with a very limited number of cases with MIC ≥ 1 mg/L in these later studies may explain these negative results.

### 3.3. Azoles

Fluconazole is the most widely used azole for the treatment of IC, being recommended as first or second line treatment [1,2,5]. Other azoles are rarely used in this setting.

Murine models of *C. albicans* IC showed that a fluconazole AUC/MIC ratio >25–100 was the best parameter in predicting outcome [27,28]. For *C. glabrata*, murine models of IC showed overall reduced efficacy of fluconazole independently from MIC results [29,30]. For fluconazole, the 24-h serum AUC is roughly equivalent to the daily dose in patients with normal body habitus and renal function.

Mechanisms of azole resistance in *Candida* spp. are multiple and complex (e.g., mutations in *ERG11* target gene, overexpression of target genes and/or drug transporters) [31], which can be associated with different levels of resistance, fitness, and virulence, and renders MIC interpretation more complex.

Several clinical studies have tried to correlate the *Candida* MICs or other composite pharmacodynamic parameters (dose/MIC, AUC/MIC) with outcome of IC (Table 2). Interpretation of these studies is confounded by heterogeneity according to the susceptibility testing method, the overall number of cases, and the rate of resistant isolates (i.e., their statistical power) and the outcome definitions (e.g., overall or attributable mortality, different composite scores of response to therapy), which could partly explain their discordant results. Most importantly, the differences in the proportion of *C. albicans* versus non-*albicans Candida* spp. (NAC) and the frequent pooled analysis of these cases represents the major confounding factor. It is also important to mention that the CLSI method and interpretive criteria have evolved over time, which precludes comparison between studies using the old CLSI method/criteria (before 2012) and those using the new ones with MIC cut-offs that are lower and closer to those from EUCAST [8,9,10,11,32].

The most recent and largest studies using the current methods (EUCAST, CLSI M38-A2, or Sensititre YeastOne^TM^) tend to confirm a CBP around 2 mg/L for *C. albicans* [33,34,35]. However, this MIC/outcome relationship is absent or less obvious in studies reporting both *C. albicans* and NAC cases [36,37]. Some studies limited to *C. glabrata* candidemia suggest that the dose/AUC or AUC/MIC ratio could be more relevant to predict efficacy for this pathogen exhibiting a wide range of fluconazole MIC and a dose-dependent response to azole therapy [37,38].

### 3.4. Echinocandins

The three echinocandins (caspofungin, anidulafungin, and micafungin) currently represent the first choice treatment of IC [1,2,5].

The parameters best predicting efficacy of echinocandins in murine models of IC were the C_max_/MIC and AUC/MIC ratios [44,45,46]. Caspofungin exhibits an important post-antifungal effect with persistent therapeutic concentrations in tissues [46]. The AUC/MIC targets were found to be similar for the three echinocandins when considering the free drug concentration (because of the high rate of protein binding of these drugs), but notably higher for *C. albicans* compared to *C. glabrata* or *C. parapsilosis* (ratios of 20 vs. 7, respectively) [47]. There is some controversy about the appropriateness of the currently recommended dosing regimens, which could not be sufficient to achieve the targeted AUC/MIC ratios, in particular, among critically-ill or neutropenic patients or in case of high body mass index [48,49,50].

For echinocandins, treatment failure has been clearly correlated to the presence of *FKS* hotspot mutations conferring non-susceptibility across all echinocandins, which is currently observed in about 5–10% of *C. glabrata* and 1–5% of *C. albicans* bloodstream isolates in US [51,52,53]. Overall, the rates of echinocandin treatment failure for candidemia due to *FKS*-mutant *Candida* spp. ranges from 60 to 90% [51,52,54,55], while the usual failure rate of echinocandins in the big trials of candidemia is around 10 to 30% [56,57,58,59].

Clinical studies of MIC/outcome correlation for echinocandins are shown in Table 3. Analyses derived from the caspofungin clinical trial database including several phase II and III trials of IC and *Candida* esophagitis did not find any correlation between echinocandin MICs and outcome [60,61]. However, these studies have been performed before the emergence of echinocandin resistance and included *Candida* isolates with a low and narrow MIC range. One study considering two clinical trials of micafungin for IC (total 493 cases) showed a correlation between clinical/mycological response and outcome according to the AUC/MIC ratio (cut-off 3000) and showed a trend towards improved mycological response for a micafungin MIC cut-off of <0.5 mg/L (*p* = 0.07) [62]. The most recent studies, including a substantial proportion of *FKS* mutant isolates and limited to *C. glabrata* (i.e., the species for which these mutations are more frequent), showed some association between MIC and outcome [51,52,55,63,64] (Table 3).

Overall, these MIC cut-offs are close to the actual CLSI CBPs, which demonstrated a good accuracy in identifying *FKS* mutant isolates [55,65]. Shields et al. showed that CLSI breakpoints of non-susceptibility for anidulafungin (≥0.25 mg/L) and micafungin (≥0.12 mg/L) were highly specific for the identification of *FKS*-mutant *C. glabrata* isolates and predicted treatment failure with 23–27% sensitivity and 89–98% specificity [55]. However, caspofungin CBPs appear as less reliable predictor of *FKS* mutations, which may be related to some methodological testing issue and interlaboratory variability of MIC results [16,55].

Whether higher echinocandin doses can overcome resistance in *FKS*-mutant strains remains a debated question with controversial results in murine models, suggesting that the effect could be strain-dependent and/or related to the type of mutation [66,67,68]. It is noteworthy that such *FKS* mutations may occur with a consequent fitness cost, which may result in decreased virulence and lower AUC/MIC ratio for efficacy in murine models [50,66,69].

In addition to the phenomenon of acquired *FKS* mutations among *C. albicans* and *C. glabrata*, decreased echinocandin susceptibility is observed in *C. parapsilosis*, which has a natural mutation at codon A661 of *FKS1.* However, echinocandin therapy demonstrated a global response of 60–90% for *C. parapsilosis* IC in large prospective comparative trials, which did not differ from that of IC due to other *Candida* spp. [56,57,58,59]. Finally, Fernandez-Ruiz et al. observed that initial echinocandin treatment had no impact on clinical failure among 194 cases of *C. parapsilosis* candidemia of a Spanish cohort [70]. One pharmacodynamic analysis showed a trend towards improved mycological response of *C. parapsilosis* IC to micafungin for an AUC/MIC ratio ≥285, which was about 7-fold lower than for *C. albicans* [62]. This might be explained by the fact that *C. parapsilosis* is considered as a less-virulent *Candida* species associated with catheter infection and for which adequate source control by catheter removal may be sufficient for clearance of the infection.

### 3.5. Current Issues/Pitfalls for Candida spp. AST

CLSI and EUCAST have now harmonized their CBPs for *Candida* spp. and azoles (Table 1). Relative robust data support the *C. albicans* CBP for fluconazole, while it seems less evident for NAC, in particular for *C. glabrata*, for which both CLSI and EUCAST abstain from a clear categorization and consider most isolates to fall in the susceptible-dose dependent or intermediate category.

For echinocandins, there is robust evidence of the association of *FKS* mutations among *C. albicans*/*glabrata* and failure of therapy. CLSI CBPs display acceptable reliability for their identification. Discrepancies between CLSI and EUCAST CBPs, which can be attributed to differences in testing methods, remain an important issue (Table 1). Microbiology laboratories should be aware to use the appropriate CBPs according to their testing method, in particular, regarding the widely used commercial kit Sensititre YeastOne^TM^, for which CLSI CBPs are recommended. Use of EUCAST criteria with this method would result in overestimation of echinocandin resistance. The role of caspofungin AST and its interpretation is still a matter of debate, and both CLSI and EUCAST recommend to rely on anidulafungin/micafungin MICs for interpretation.

The role and interpretation of AST in NAC other than *C. glabrata* still remain unclear. In particular, there is currently no established CBPs for emerging *Candida auris*, which exhibit a large variability of MIC values and a remarkable ability to rapidly induce resistance to all three antifungal drug classes [71,72]. These isolates should be tested and results interpreted using CBPs derived from other *Candida* spp. [72], but their actual relevance should be investigated in clinical studies.

### 3.6. Invasive Aspergillosis (IA)

IA and invasive mold infections in general are challenging for the assessment of CBPs because of their frequent lack of microbiological documentation and therefore of MIC data. Over 50% of IA are nowadays diagnosed on the basis of a positive galactomannan only, without recovery of the mold in culture [73]. Currently, AST is not routinely recommended for all *Aspergillus* isolates [4]. However, emerging pan-azole resistance among *A. fumigatus* makes that this practice is encouraged for documented IA [4] and raises the need to better correlate the results of AST testing with response to therapy.

### 3.7. Amphotericin B

Amphotericin B (AMB) formulations are still widely used for the treatment of IA, in particular, as initial pre-emptive therapy and/or in the setting of breakthrough to azole prophylaxis [3,4,74]. With the exception of *A. terreus* exhibiting innate resistance to AMB, and to a lesser extent *A. flavus*, the other common pathogenic *Aspergillus* spp. including *A. fumigatus* exhibit low and narrow ranges of MICs [75]. Acquired AMB resistance among *A. fumigatus* seems to be extremely rare, as it is associated with fitness cost, and its mechanisms are not well understood [76].

The C_max_/MIC was the best parameter predicting response to therapy in mice with an optimal ratio at 2.4 [77]. All formulations of AMB induce a dose-dependent response that can also be linked to the AUC/MIC, but there is very distinct concentration-response and AUC-response profiles between the three AMB formulations (deoxycholate, liposomal, and lipid complex) [78,79].

Pk/Pd murine models of IA assessing the efficacy of AMB against *Aspergillus* spp. with different MIC levels provided controversial results, some suggesting a MIC/outcome correlation and others not [80,81,82].

Clinical studies are also scarce with very limited number of cases, and include a mixture of *Aspergillus* spp. (including *A. terreus*) [83,84] (Table 4). Only one study identified a cut-off of ≥2 mg/L to predict failure, which may be actually more species-related (*A. terreus*/*flavus*) than MIC-related.

### 3.8. Azoles

Voriconazole is the first-line treatment of IA, while the novel triazoles isavuconazole and posaconazole represent alternative therapeutic options [3,4]. *A. fumigatus*, the most common cause of IA, and other pathogenic *Aspergillus* spp. exhibit low and narrow MIC ranges to these drugs [95,96,97].

Acquired azole resistance among *A. fumigatus* is an emerging issue, which is currently observed at variable incidence around the world, from <1% to 15% (e.g., in the Netherlands) [98,99]. Isolates harboring the typical TR_34_/L98H mutation in the *cyp51A* gene usually display pan-azole resistance with high MIC values (≥8 µg/mL for voriconazole) [98,100]. Other mutations have been described, with variable levels of resistance [101]. One study suggests that the proportion of *A. fumigatus cyp51A*-mutant isolates becomes significant (≥10%) among isolates with a MIC ≥2 mg/L for voriconazole and ≥0.5 mg/L for posaconazole [100]. Acquired azole resistance is much less frequently reported among the other common pathogenic *Aspergillus* spp. (*A. flavus*, *A. niger*, *A. terreus*), but could be an underrecognized issue [102].

In murine Pk-Pd models of IA, the AUC/MIC ratio is the most reliable index for exposure-response analyses [103,104,105,106,107,108]. A standard dosing regimen is expected to achieve appropriate exposure for efficacy against *A. fumigatus* isolates with a MIC ≤1 or 2 mg/L for voriconazole, ≤0.25 mg/L for posaconazole and ≤2 mg/L for isavuconazole [103,104,105,106,107,108]. These values are close to the established epidemiological cut-off values (ECVs) [95,96,97]. Pk-Pd murine models showed that the efficacy of triazoles was significantly decreased against *cyp51A*-mutant isolates, but could be improved with escalating doses [104,105,106,107,108].

Most clinical pharmacodynamic studies of azoles and IA have assessed the correlation between pharmacologic parameters and outcome, instead of MIC/outcome. Effectively, MIC documentation is lacking in many IA cases and, before the recent emergence of azole resistance, most *A. fumigatus* isolates exhibited a low and narrow range of MICs. On the contrary, azole drugs (mainly voriconazole and to some degree posaconazole) display important pharmacokinetic inter- and intra-individual variability. As the trough concentration (C_trough_) correlates with AUC [109], it can be easily monitored in clinical practice. These studies showed an association between a voriconazole C_trough_ ≥1 to 2 mg/L, or a C_trough_/MIC ratio >2 to 10 and therapeutic success, but are hampered by their very heterogeneous dataset including all types of IFI (usually a mix of IA and IC) [90,91,92,93,94]. These results are however supported by an in vitro model of IA using cell culture of the human alveolus and mimicking voriconazole pharmacokinetics in humans suggesting that a C_trough_/MIC ratio of 1 to 2 is a predictor of success [104].

Studies correlating MIC/outcome and limited to IA are scarce, but are actually needed with the raising concern of emerging azole resistance. Their results are controversial (Table 4), which is mainly due to the low proportion of *cyp51A* mutant isolates (affecting their actual statistical power) and the confounding factor of non-azole drugs (in particular, use of amphotericin B among patients infected with azole-resistant isolates, which represents an important bias in outcome analyses). The most relevant results have been recently reported in a large Dutch cohort of IA including a high proportion of *cyp51A* mutant isolates and in which the majority of patients have received initial voriconazole therapy [87]. In this study, a voriconazole MIC cut-off >2 mg/L was associated with a higher mortality rate (47% vs. 24% for MIC ≤2 mg/L, *p* = 0.02).

The efficacy of increased azole doses for the treatment of *cyp51A*-mutant isolates is still an open question. One study reported an overall good response to high-dose posaconazole (targeted C_trough_ > 3 mg/L) in a series of 7 IA with resistant isolates (MIC ≥ 16 mg/L, most of them with documented *cyp51A* mutations) [110].

In addition to acquired resistance among *A. fumigatus*, some rare cryptic *Aspergillus* spp. (e.g., *A. calidoustus*, *A. lentulus*) intrinsically exhibit decreased susceptibility to azoles [111,112,113]. Limited data are available regarding the efficacy of azoles against these cryptic *Aspergillus* spp. Voriconazole displayed similar efficacy than amphotericin B against *Aspergillus calidoustus* in a *Galleria mellonella* model of infection and in an retrospective outcome analysis of clinical cases, while this species usually exhibits high MICs to voriconazole (4–8 mg/L) [112,114].

### 3.9. Echinocandins

Echinocandins only have fungistatic activity against *Aspergillus* spp. and are considered as second-line or third-line therapy of IA in case of intolerance or failure of azoles or amphotericin B [3,4]. In vitro, the fungistatic effect is observed at relatively low concentrations with a very narrow range of minimal effective concentrations (MEC). Variable ECVs have been reported across studies, which actually reflects variations in methods and reading interpretation [115,116,117]. Acquired echinocandin resistance among *Aspergillus* spp. can result from mutation in the target gene (*FKS1*) and seems to be an extremely rare event as the cost of such mutations may be an important loss of fitness [118,119]. The effect of echinocandins against *A. fumigatus* is dose-dependent, but caspofungin may have a paradoxical effect in vitro (i.e., initial dose-dependent effect followed by loss of activity at increasing concentrations), which was also observed in murine models [120,121,122]. In a murine Pk/Pd model of IA, the caspofungin C_max_/MEC ratio appeared as the most reliable indicator with optimal efficacy at 10–20 [122].

There are no data correlating echinocandins MICs and IA outcome. Considering the narrow MIC distribution and the extreme rarity of acquired echinocandin resistance among *A. fumigatus*, it is unlikely that such analyses would provide any relevant conclusions.

### 3.10. Current Issues/Pitfalls for Aspergillus spp. AST

The MIC interpretation and CBP definitions for *Aspergillus* spp. continue to be a matter of uncertainty. While EUCAST has defined clinical CBPs for azoles and amphotericin B, CLSI abstains from a susceptibility/resistance categorization and only provides ECVs (Table 1). Although there is good agreement between these CBP/ECV values, some issues should be outlined.

For amphotericin B, the very narrow interval of categorization proposed by EUCAST (i.e., ≤1 mg/L susceptible, 2 mg/L intermediate and ≥4 mg/L resistant) is concerning. Effectively, these CBPs fall within the narrow range of MIC distribution of *A. fumigatus* (1 to 4 mg/L), and a single-dilution difference could be not significant, considering the acceptable technical variability of ± two dilutions for AST testing of molds. The concept of defining CBPs for amphotericin B and *A. fumigatus* is debated in the absence of established and clinically-relevant mechanisms of resistance to this drug. MIC differences in this setting are more likely to reflect technical variability.

For azoles, there is relatively good evidence to suggest that voriconazole therapy is appropriate for isolates with MIC ≤2 mg/L, provided appropriate monitoring of voriconazole concentrations for a targeted C_trough_ ≥2 mg/L. Presence of *cyp51A* mutation actually represents the most reliable predictor of failure of azole therapy and these isolates usually exhibit high MICs (≥8 mg/L), which can be easily identified. Because therapeutic options of azole-resistant IA are very limited, the potential role of higher azole doses for a dose-dependent effect should be further investigated. However, the relatively narrow therapeutic range of azoles, especially voriconazole, represents a limitation to this approach.

For echinocandins, current data do not support a recommendation for AST and MIC interpretation. The fact that widely used commercial kits (e.g., Sensititre YeastOne^TM^) include echinocandin testing in their panel could be confusing for the clinicians, in particular, because of the low MICs of *Aspergillus* spp., which actually correspond to MECs and cannot be translated in good efficacy.

Regarding the testing methods, there is an increased use of more convenient and less time-consuming approaches, such as E-test. It should be outlined that E-test for *Aspergillus* spp. usually provide lower MICs (in particular for posaconazole) compared to those obtained by standard CLSI or EUCAST microbroth dilution methods [14].

### 3.11. Non-Aspergillus Invasive Mold Infections (NAIMIs)

NAIMIs consist mainly of invasive mucormycosis (IM), followed by disseminated fusariosis and scedosporiosis. Fungal pathogens causing these infections share the characteristics of resistance or decreased susceptibility to at least one or two of the currently available antifungal drug classes and are considered as “difficult to treat”. Some of them have a highly predictable antifungal susceptibility profile (e.g., *Scedosporium* spp.), but others exhibit a wide range of MIC distribution. This is notably the case for species of the order *Mucorales* (causal agents of IM) with respect to the broad-spectrum azoles (posaconazole and isavuconazole) and for *Fusarium* spp. (causal agents of fusariosis) and voriconazole [123,124,125]. To a lesser extent, variable MICs to amphotericin B are also observed for these mold species [124,125]. Despite these observations, mechanisms of acquired resistance remain largely unknown among non-*Aspergillus* molds.

Murine models are scarce and have failed to demonstrate a correlation between MICs and outcome for IM and fusariosis [126,127,128]. This lack of association could be partly explained by the overall poor efficacy of antifungals in these models and/or by the narrow MIC ranges of the tested strains. For *Scedosporium apiospermum* infection, one study in mice suggested a correlation between voriconazole MICs >2 mg/L and failure [129].

Clinical pharmacodynamic studies for NAIMIs are quasi-absent because of the rarity of the disease and the frequent lack of positive culture results, in particular for IM. One retrospective small-series of NAIMIs (consisting mainly of mucormycosis) suggested a correlation between amphotericin B MIC >0.5 mg/L and failure of therapy [130]. For fusariosis, voriconazole, despite its wide MIC distribution and frequent high MIC values (≥8 mg/L), was found to be equally effective than lipid formulations of amphotericin B (exhibiting overall lower MICs) [131,132].

Most importantly, the studies reporting factors associated with NAIMI outcomes demonstrated that non-pharmacologic factors, such as the type and duration of immunosuppression, the localization and extension of disease, the timing of appropriate antifungal therapy, and surgical interventions actually represented the best predictors of outcome [132,133,134,135].

### 3.12. Current Issues/Pitfalls for Non-Aspergillus Molds AST

There is no recommendation of AST testing for non-*Aspergillus* molds as MICs are considered as unreliable predictors of outcome and actual mechanisms of acquired resistance are unknown. Nonetheless, a wide range of MIC distribution is frequently observed, in particular for *Mucorales* and posaconazole/isavuconazole, and for *Fusarium* spp. and voriconazole. A better understanding of the causes and actual significance of these MIC variations is needed.

## 4. Conclusions and Perspectives

The assessment of CBPs for fungi remains a difficult task, as illustrated by the ongoing processes of revision and harmonization of the CLSI and EUCAST criteria. AST is recommended for *Candida* spp. (azoles and echinocandins) and *Aspergillus fumigatus* (azoles) isolated in invasive diseases. Notably, the mechanisms of resistance for these antifungals/pathogens are well-described (i.e., acquired mutations) and the performance of CBPs or ECVs seems acceptable for their identification with a high probability of failure of therapy. For *Candida*/echinocandins and *Aspergillus*/azoles, a single mechanism of resistance (i.e., mutations in hotspots of the target gene) seems to be by far the most clinically relevant. Therefore, genotypic rather than phenotypic characterization of the isolates might be a most reliable indicator to guide therapeutic choices. Direct identification of these mutations by PCR tools has also the considerable advantage to provide more rapid results compared to AST that requires initial positive cultures and subcultures resulting in important delays. Several PCR kits have been developed for the rapid and accurate identification of *FKS* mutations (for *Candida* spp.) or *cyp51A* mutations (for *Aspergillus* spp.) on cultures or directly on the clinical samples [136,137,138]. Albeit promising, these molecular diagnostic tools might be more costly with limited availability. Therefore, AST is expected to remain an important pillar for resistance detection.

Because of the heterogeneity of AST methods, the area of technical uncertainty and the difficulties in MIC interpretation (as mentioned above), input by mycology experts is required for the interface between the laboratory and beside in order to avoid misinterpretation. Indications for AST should also be clearly delineated and each request of fungal AST falling outside these indications should be carefully evaluated and discussed with the clinicians. Systematic AST may have an interest for epidemiological purposes and monitoring of resistance, but can also be confusing for the clinicians in the absence of expert guidance for interpretation.

The significance of MIC results should always be interpreted in a global context, taking into account the multiple parameters that can affect outcome, including host factors, the pathogenesis of the disease, the drug bioavailability, and non-pharmacologic interventions. Lack of response to therapy despite a susceptible fungal pathogen and an appropriate therapy remains a relatively frequent situation. On the contrary, a favorable response despite apparently high MIC of the fungus to the ongoing antifungal drug can also be observed. Several mechanisms that can influence clinical response (Figure 1) and that are not reflected by MIC may occur. For instance, acquired mutations of resistance are often associated with a fitness loss and decreased virulence. Heteroresistance can also occur with an undetected subpopulation of resistant clones despite apparent low MIC. Such phenomenon might be observed in patients with sanctuary sites and poor drug penetration (e.g., intra-abdominal IC, aspergilloma, or large necrotic mass in IA or IM).

Finally, several priority axes of research in this complex topic can be highlighted. On the molecular laboratory side, there is a need to improve our understanding of the clinically-relevant mechanisms of acquired resistance, in particular regarding azole resistance other than *cyp51A* mutations in *Aspergillus* and the still largely unknown mechanisms of resistance in non-*Aspergillus* molds. On the clinical microbiology laboratory side, efforts should be pursued for more uniform practices and interpretive criteria. On the clinical side, studies of MCI/outcome correlation should be undertaken in large multicenter cohorts of relatively homogenous patient populations, allowing for integration of potential confounding factors in multivariate analyses.

## Figures and Tables

**Table 1:** Comparison of CLSI and EUCAST clinical breakpoints of antifungal drugs for most relevant *Candida* and *Aspergillus* spp. according to CLSI and EUCAST.

Species	AMB		FLC		VRC		POS		CAS		AND		MCF	
*C. albicans*	ND	1	2 (8)	2 (8)	0.12 (1)	0.06 (0.5)	ND	0.06	0.25 (1)	ND	0.25 (1)	0.03	0.25 (1)	0.016
*C. tropicalis*	ND	1	2 (8)	2 (8)	0.12 (1)	0.12 (0.5)	ND	0.06	0.25 (1)	ND	0.25 (1)	0.06	0.25 (1)	ND
*C. parapsilosis*	ND	1	2 (8)	2 (8)	0.12 (1)	0.12 (0.5)	ND	0.06	2 (8)	ND	2 (8)	0.002 (8)	2 (8)	0.002 (4)
*C. glabrata*	ND	1	32 (SDD) (64)	0.002 (64)	ND	ND	ND	ND	0.12 (0.5)	ND	0.12 (0.5)	0.06	0.06 (0.25)	0.032
*C. krusei*	ND	1	(R)	(R)	0.5 (2)	ND	ND	ND	0.25 (1)	ND	0.25 (1)	0.06	0.25 (1)	ND
*A. fumigatus*	ND	1 (4)	(R)	(R)	ND	1 (4)	ND	0.125 (0.5)	ND	ND	ND	ND	ND	ND
*A. flavus*	ND	ND	(R)	(R)	ND	ND	ND	ND	ND	ND	ND	ND	ND	ND
*A. niger*	ND	1 (4)	(R)	(R)	ND	ND	ND	ND	ND	ND	ND	ND	ND	ND
*A. terreus*	(R)	(R)	(R)	(R)	ND	ND	ND	0.125 (0.5)	ND	ND	ND	ND	ND	ND

Clinical breakpoints (CBPs) of the Clinical and Laboratory Standards Institute (CLSI, left column) and European Committee on Antimicrobial Susceptibility Testing (EUCAST, right column). The numbers indicate the CBP [mg/L] for the distinction between susceptible “S” (≤the indicated value) vs. non-susceptible. If an intermediate “I” or “susceptible dose-dependent” (SDD) category has been defined, the resistance “R” cut-off (≥the indicated value) is mentioned in brackets. ND: no defined CBP (insufficient evidence), (R): the species is considered as intrinsically resistant (susceptibility testing not recommended).

**Table 2:** Clinical studies correlating fluconazole minimal inhibitory concentrations (MIC) and outcome of invasive candidiasis (IC).

Study (First Author, Year, Reference)	N Cases	Type of IC (Patients)	*Candida* spp.	AST Method	Outcome Indicator	Correlation MIC/Outcome ^1^	MIC Cut-Off for Success
Rex (1995)	232	Candidemia (non-neutropenic)	*C. albicans* 56% NAC 44%	NCCLS (M27-P)	Clinical response ^2^	No	-
Lee (2000) [39]	32	All IC (mainly non-neutropenic)	*C. albicans* 53% NAC 47%	NCCLS (M27-A)	Clinical response ^2^	Trend	MIC ≤ 8 mg/L
Kovacicova (2000) [40]	161	Candidemia (mixed)	*C. albicans* and NAC (NS)	Disk diffusion (some E-test)	Attributable mortality	Yes	“susceptible”
Clancy (2005) [41]	32	Candidemia (mixed)	*C. albicans* 37% NAC 63%	NCCLS (M27-A)	Clinical response ^2^	Yes	MIC ≤ 8 mg/L Dose/MIC > 50
Pai (2007) [42]	77	Candidemia (non-neutropenic)	*C. albicans* 64% NAC 36%	CLSI (M27-A2)	Overall mortality (hospital discharge)	Trend (MIC alone) Yes (ratios)	MIC ≤ 8 mg/L Dose_wn_/MIC > 12 AUC/MIC > 55
Rodriguez-Tudela (2007) [34]	126	Candidemia (mainly non-neutropenic)	*C. albicans* 58% NAC 42%	EUCAST	Clinical response ^2^	Yes	MIC ≤ 2 mg/L Dose/MIC ≥ 33.5
Baddley (2008) [43]	84	Candidemia (mainly non-neutropenic)	*C. albicans* 44% NAC 56%	CLSI (M27-A2)	Overall mortality (week 6)	Yes	MIC ≤ 32 mg/L AUC/MIC ≥ 11.5 Dose/MIC ≥ 12.5
Eschenauer (2013) [38]	122	Candidemia (mainly non-neutropenic)	*C. glabrata*	CLSI (M27-A3)	Clinical response (day 14)	Yes	Dose/MIC > 12.5
Van Hal (2014) [35]	217	Candidemia (mainly non-neutropenic)	*C. albicans*	SYO	Attributable mortality (day 30)	Yes	MIC ≤1 mg/L
Brosh-Nissimov (2015) [33]	75	Candidemia (mainly non-neutropenic)	*C. albicans* 48% NAC 52%	E-test	Overall mortality (day 30)	Yes (*C. albicans*) No (NAC)	MIC ≤ 2 mg/L Dose/MIC > 400 AUC/MIC > 400 -
Fernandez-Ruiz (2017) [36]	257	Candidemia (mainly non-neutropenic)	*C. albicans* 54% NAC 46%	CLSI (M27-A3) EUCAST	Clinical response ^2^	Trend	MIC ≤ 0.25 mg/L (CLSI)
Ghanem-Zoubi (2019) [37]	158	Candidemia (mainly non-neutropenic)	*C. albicans* 42% NAC 58%	E-test Vitek-2	Overall mortality (day 30) Clinical response (day 14)	No (all species) Trend (*C. glabrata*) No	- AUC/MIC ≥ 400

IC: invasive candidiasis, AST: antifungal susceptibility testing, MIC: minimal inhibitory concentration, NAC: non-*albicans Candida* spp., NCCLS: National Committee for Clinical Laboratory Standards, CLSI: Clinical and Laboratory Standards Institute (former NCCLS), EUCAST: European Committee on Antimicrobial Susceptibility testing, SYO: Sensititre YeastOne^TM^, AUC = area under the time-concentration curve, Dose_wn_ = dose normalized to weight, NS: not specified. ^1^ Assessment of correlation MIC/outcome: Yes (*p* < 0.05)/Trend (*p* < 0.2)/No (*p* ≥ 0.2). ^2^ Clinical response was usually assessed with composite parameters including clinical response, microbiological cure, overall mortality, relapsing candidemia.

**Table 3:** Clinical studies correlating echinocandin minimal inhibitory concentrations (MIC) and outcome of invasive candidiasis (IC).

Study (First Author, Year, Reference)	N Cases	Type of IC (Patients)	*Candida* spp.	Echinocandin	AST Method	Outcome Indicator	Correlation MIC/Outcome ^1^	MIC Cut-Off for Success
Kartsonis (2005) [60]	114	All IC (~70% non-neutropenic)	*C. albicans* 38% NAC 62%	CSF	NCCLS (M27-A)	Clinical response ^2^	No	-
Andes (2011) [62]	493	All IC (~90% non-neutropenic)	*C. albicans* 44% NAC 56%	MCF	CLSI (M27-A)	Clinical response ^2^	Yes	AUC/MIC > 3000
Shields (2013) [55,64]	66	All IC (NS)	*C. glabrata*	CSF (N = 63)	CLSI (M27-A3)	Clinical response ^2^	Yes Trend Trend	AND MIC ≤ 0.12 mg/L MCF MIC ≤ 0.06 mg/L CSF MIC ≤ 0.5 mg/L
Alexander (2013) [51]	155	Candidemia (mainly non-neutropenic)	*C. glabrata*	CSF and MCF	CLSI (M27-A3)	Clinical response ^2^ (day 10)	No	-
Farmakiotis (2014) [63]	93	Candidemia (~60% non-neutropenic)	*C. glabrata*	All (NS)	CLSI (M27-A2)	Overall mortality (day 28)	Yes	CSF MIC ≤ 0.25 mg/L
Beyda (2014) [52]	57	Candidemia (NS)	*C. glabrata*	MCF	SYO	Clinical response ^2^ (day 14)	Yes	CSF MIC ≤ 0.12 mg/L

IC: invasive candidiasis, AST: antifungal susceptibility testing, MIC: minimal inhibitory concentration, NAC: non-*albicans Candida* spp., NCCLS: National Committee for Clinical Laboratory Standards, CLSI: Clinical and Laboratory Standards Institute (former NCCLS), SYO: Sensititre YeastOne^TM^, AND: anidulafungin, CSF: caspofungin, MCF: micafungin, NS: not specified. ^1^ Assessment of correlation MIC/outcome: Yes (*p* < 0.05)/Trend (*p* < 0.2)/No (*p* ≥ 0.2). ^2^ Clinical response was usually assessed with composite parameters including clinical response, microbiological cure, overall mortality, relapsing candidemia.

**Table 4:** Clinical studies correlating antifungal minimal inhibitory concentrations (MIC) and outcome of invasive aspergillosis (IA).

Study (First Author, Year, Reference)	N Cases	Type of IA (Patients)	*Aspergillus* spp.	AF Drug	AST Method	Outcome Indicator	Correlation MIC/Outcome ^1^	MIC Cut-Off for Success
**Azoles MIC/outcomes**								
Heo (2017) [85]	107	Proven/probable ^2^ (HEM)	*A. fumigatus* 49% (no *cyp51A* mutations ^3^) Others: 51%	VRC (44%) L-AmB (54%)	CLSI (M38-A2)	Overall mortality (day 42)	No	-
Andes (2019) [86]	22	Proven/probable ^2^ (mainly HEM)	NS	VRC	CLSI (M38-A2) EUCAST	Overall mortality (day 42) Clinical response	No	-
Lestrade (2019) [87]	196	Proven/probable or putative ^2^ (HEM 53%)	*A. fumigatus* (32 with *cyp51A* mutations ^3^)	VRC (79%) ^4^	EUCAST	Overall mortality (day 42 and 90)	Yes	MIC ≤ 2 mg/L
Andes (2019) [86]	49	Proven/probable ^2^ (mainly HEM)	NS	ISA	CLSI (M38-A2) EUCAST	Overall mortality (day 42) Clinical response	No	-
**Amphotericin B MIC/outcomes**								
Lass-Flörl (1998) [83]	29	Proven/probable ^2^ (HEM)	*A. flavus* (12), *A. terreus* (9), *A. fumigatus* (8)	d-AmB	NCCLS (M27-P)	Overall mortality	Yes	MIC ≤ 1 mg/L
Lionakis (2005) [84]	18	Proven/probable ^2^ (mainly HEM)	*A. terreus* 33% Others: NS	L-AmB	NCCLS (M38-A)	Clinical response (day 14)	No	-

MIC: minimal inhibitory concentration, IA: invasive aspergillosis, AF: antifungal, AST: antifungal susceptibility testing, HEM: hematologic cancer patients, VRC: voriconazole, ISA: isavuconazole, d-AmB: deoxycholate amphotericin B, L-AmB: lipid formulation of amphotericin B, EUCAST: European Committee on Antimicrobial Susceptibility testing, NCCLS: National Committee for Clinical Laboratory Standards, CLSI: Clinical and Laboratory Standards Institute (former NCCLS), NS: not specified. ^1^ Assessment of correlation MIC/outcome: Yes (*p* < 0.05)/Trend (*p* < 0.2)/No (*p* ≥ 0.2). ^2^ IA classification: proven/probable according to EORTC-MSG criteria (or equivalent) for immunocompromised patients [88], proven/putative according to the AspICU algorithm for intensive care units patients [89]. ^3^ No mutations known to be associated with azole resistance (in particular TR_34_/L98H or TR_46_/Y121F/T289A). ^4^ Voriconazole was the first-line AF in 79% of cases. N.B.: Only studies limited to IA are mentioned in this Table. Pharmacodynamics studies including a mix of different invasive fungal infections [90,91,92,93,94] have not been included in this Table.

## Data Availability

Not applicable.
